# Bimanual Force Production at 90-Degree Relative Phase with Lissajous Feedback

**DOI:** 10.3390/brainsci16050462

**Published:** 2026-04-25

**Authors:** Naoki Hamada, Shiho Fukuda, Han Gao, Hitoshi Oda, Hiroshi Kunimura, Taku Kawasaki, Koichi Hiraoka

**Affiliations:** Graduate School of Rehabilitation Science, Osaka Metropolitan University, 2-1-132 Morinomiya, Joto-ku, Osaka 536-8525, Japan; w12relax@gmail.com (N.H.); shiho7755@outlook.jp (S.F.); gh1823020@outlook.com (H.G.); hito_pt23@yahoo.co.jp (H.O.); hiroshihayabusa821@yahoo.co.jp (H.K.); t.kawasaki@hakuho.ac.jp (T.K.)

**Keywords:** bimanual coordination, relative phase, motor binding, tactile stimulation, Lissajous plot feedback

## Abstract

**Background/Objectives**: Bimanual movements with a 90° relative phase are typically unstable but can be facilitated by Lissajous visual feedback, which integrates the movements of the two hands into a single visual representation. We examined whether such visual integration leads to a unified sensorimotor representation by testing whether unilateral tactile stimulation suppresses motor output bilaterally during bimanual force production. **Methods**: Fifteen healthy participants produced rhythmic bimanual index finger flexion with a 90° relative phase under two feedback conditions: Lissajous feedback and individual visual feedback. In each trial, vibrotactile stimulation was applied to either hand or not applied at one of four phases of the force cycle. Force trajectory error and post-stimulus electromyographic (EMG) activity of the first dorsal interosseous muscle were analyzed. **Results and Discussion**: Lissajous feedback reduced force trajectory error compared with individual feedback. Tactile stimulation did not produce bilateral suppression of motor output. This indicates that visual integration of bimanual movements does not lead to global bilateral suppression of motor output induced by unilateral tactile stimulation. A significant reduction in post-stimulus EMG amplitude was observed only when the right hand was stimulated during one phase of the Lissajous feedback task. This suppression may reflect the unmasking of the tactile stimulus-induced inhibition within sensorimotor processes in the left hemisphere when visual feedback of the two hands is merged into a single representation.

## 1. Introduction

Rhythmic bimanual movement with a 90-degree relative phase, corresponding to a 1/4-cycle delay between the two hands, is unstable and difficult to perform [1,2,3,4]. The difficulty in producing bimanual movement with a 90-degree relative phase can be attributed to two main factors. First, phase attraction draws coordination between the limbs toward stable patterns such as in-phase and anti-phase, a phenomenon referred to as intrinsic dynamics [5]. Second, this instability is also associated with the activation of non-homologous muscles through crossed and uncrossed cortical pathways [6].

When visual feedback of the left-hand target movement is provided along the horizontal axis and that of the right hand along the vertical axis with a 90-degree relative phase, the resulting trajectory becomes circular, a representation known as Lissajous plot feedback [7,8,9,10,11]. This Lissajous feedback facilitates smooth execution of bimanual movement with a 90-degree relative phase [7,11,12]. The process underlying this improvement is the integration of two visual streams into a single Gestalt [13,14]. Through this transformation, the target becomes easier to understand. Recently, Lissajous feedback has been frequently used to improve the performance of bimanual movements [15,16,17].

Such visual transformations have been shown to facilitate the integration of the two limb movements into a unified motor representation, a phenomenon referred to as motor binding [14]. In sensorimotor control, motor commands and sensory feedback are integrated to form a coherent representation of ongoing movements [18]. Somatosensory signals from one limb can influence perceptual and motor processing in the contralateral limb [19], indicating interlimb somatosensory interactions. This suggests that sensory processing during bimanual movement is not strictly independent across the two limbs but may be integrated within a shared sensorimotor framework during motor binding. When representations of two hand movements are merged into a single motor representation (i.e., motor binding), the associated somatosensory processing may also be organized at the level of this global representation rather than at the level of individual limbs [14,20]. Thus, the aim of the present study was to determine whether motor binding induced by Lissajous visual feedback extends to sensorimotor inhibitory processes during bimanual coordination.

Corticospinal excitability of hand muscles is suppressed by electrical stimulation of the ipsilateral median nerve when delivered approximately 25 ms prior to transcranial magnetic stimulation, a phenomenon referred to as short-latency afferent inhibition (SAI) [21,22]. Tactile stimulation of the finger suppresses primary motor cortical activity in the first dorsal interosseous (FDI) muscle [23]. In a motor binding situation in which the bimanual motor systems operate as a single representation, we would expect that unimanual tactile stimulation induces the global suppression of motor output in both hands. If the two hands are controlled under a single global representation during bimanual motor output with the Lissajous feedback, such sensorimotor processes may operate as a single representation rather than independently for each limb. Consequently, tactile stimulation of one hand would suppress motor output in both hands. If this hypothesis is true, the Lissajous feedback task would produce inhibition of the EMG amplitude in both the stimulated and non-stimulated hands, reflecting a global motor representation during motor binding. Statistically, this prediction corresponds to a task × stimulated hand interaction, with bilateral inhibition of the EMG amplitude induced by the unilateral tactile stimulus of the hand expected specifically during the Lissajous feedback task.

## 2. Materials and Methods

### 2.1. Participants

We recruited healthy adult participants via website. People with a history of neurological or musculoskeletal disorders were excluded. No exclusion criteria were applied regarding gender or handedness. Fifteen healthy adults (fourteen males and one female) aged 34.6 ± 10.6 years participated in this study. One participant was left-handed, and fourteen participants were right-handed according to the Edinburgh Handedness Inventory [24]. The sample size was determined using an a priori power analysis (G*Power 3.1) to detect a main effect of tactile stimulation in an ANOVA design. A large effect size (f = 0.4) was assumed based on Cohen’s conventions for behavioral sciences [25]. With an alpha level of 0.05 and a desired power of 0.80, the required sample size was estimated to be 12 participants. We recruited 15 participants to ensure sufficient statistical power. Written consent was obtained from all participants. The experiment was conducted according to the Declaration of Helsinki and was approved by the ethics committee of Osaka Metropolitan University.

### 2.2. Apparatus

The participants were seated in front of a table. The forearms were placed on the table with the palms facing downward. Earmuffs were placed over the ears to minimize the influence of auditory input. A display, showing target force circles and force circles indicating index finger force, was placed 1 m in front of the participants. The diameter of each circle on the display was 10 mm. A load cell measuring the index finger flexion was placed under each index fingertip. Devices providing the vibratory stimulus with a frequency of 150 Hz (Mini vibration motor 2.0 mm, Seeds Studio, Tokyo, Japan) were placed on the dorsal hand at the third metacarpals. The vibration device was driven by a constant 5 V direct current. Prior to each experiment, the participant confirmed that the vibration was clearly perceivable and did not induce discomfort. The same voltage of the direct current activating the device was used for all participants. Thus, the intensity of the tactile stimulus was consistent across the participants. The signals from the load cells were amplified by strain amplifiers (DPM-751A, Kyowa Dengyo, Tokyo, Japan). Electrodes recording the electromyographic (EMG) activity in the FDI were placed over the belly of the muscles with the method of the belly-tendon montage. The EMG signals were amplified by an amplifier (MEG-5200; Nihon Kohden, Tokyo, Japan) with passband filters of 15 Hz–1 kHz. Analog signals from the EMG amplifiers were digitized using an A/D converter (PowerLab/8SP and 2sp; ADInstruments, Colorado Springs, CO, USA) at a sampling rate of 1 kHz, and the digitized signals were stored in a personal computer.

### 2.3. Sensitivity of Force Circles

Before the experiment, participants pressed each load cell for 6 s at maximal effort to estimate maximal force (MF). Based on this measurement, the sensitivity of the force circles on the display was adjusted. The position 200 pixels to the left of the horizontal center of the display indicated 0% MF of the left index finger flexion. The position 200 pixels to the right of the horizontal center of the display indicated 25% MF of the left index finger flexion. The position 200 pixels below the vertical center of the display indicated 0% MF of the right index finger flexion. The position 200 pixels above the vertical center of the display indicated 25% MF of the right index finger flexion.

### 2.4. Lissajous Feedback Task

**Figure 1:**
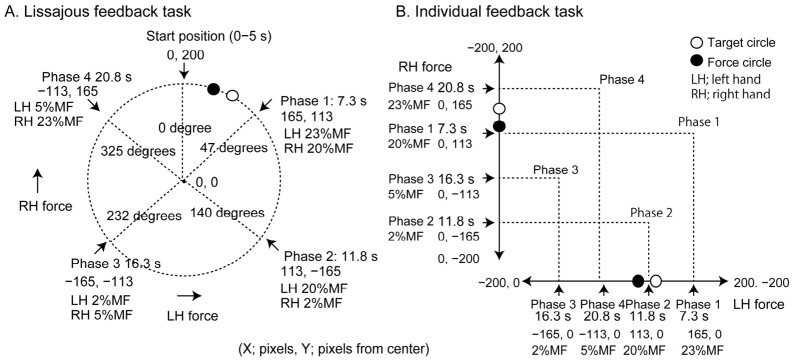
Target trajectories of the motor tasks used in the experiment. Lissajous feedback task, in which the target circle moved along a circular trajectory (**A**). Individual feedback task, in which the target forces of the left and right index fingers are indicated by circles moving vertically and horizontally, respectively (**B**).

The target movement trajectory in the Lissajous feedback task is shown in Figure 1A. Each trial consisted of a 5-s start phase followed by a 17.5-s movement phase (one cycle). Thus, the total duration of one trial was 22.5 s. The duration of one cycle (17.5 s) was chosen to ensure slow and continuous force modulation while avoiding fatigue. The long cycle duration decreases the frequency of the corrective submovements and allows the stable production of the required 90° relative phase between the hands. In our preliminary trials, this duration was suitable for the participants to perform the task without great difficulty.

During the start phase, the target remained 200 pixels above the center of the display (0, 200) for 5 s. During this phase, participants pressed the load cells to align the force circle with the target circle. Under this condition, left index finger flexion was 12.5% MF and right index finger flexion was 25% MF. After the start phase, the target circle moved along an arc at a constant velocity. The arc was centered at the display center, and its diameter was 200 pixels. The movement direction was clockwise. Participants pressed the load cells to keep the force circle aligned with the target circle while the target was present. Each trial consisted of one cycle.

### 2.5. Individual Feedback Task

The target movement trajectories of the individual feedback task are shown in Figure 1B. A target circle (red) moving vertically indicated the right index finger flexion force. Upward movement of this circle indicated an increase in the target force of the right index finger flexion. A force circle (open) moving vertically represented the actual right index finger force. Another target circle (blue) moving horizontally indicated the left index finger flexion force. Rightward movement of this circle indicated an increase in the target force of the left index finger flexion. A force circle (open) moving horizontally represented the actual left index finger force. The target circles moved at a constant velocity after the start phase. During the start phase, the red circle (right-hand force target) was presented 200 pixels below the center of the display (0, −200), corresponding to 0% MF of the right hand flexion force. The blue circle (left-hand force target) was presented 200 pixels to the left of and 200 pixels below the center of the display (−200, −200), corresponding to 0% MF of the left hand flexion force. After the start phase, the red target circle moved vertically and the blue target circle moved horizontally. A trial terminated after one force cycle. The same cycle duration (17.5 s) was used as in the Lissajous feedback task in the individual feedback task to ensure that the temporal demands were comparable between the tasks.

### 2.6. Tactile Stimulation

**Figure 2:**
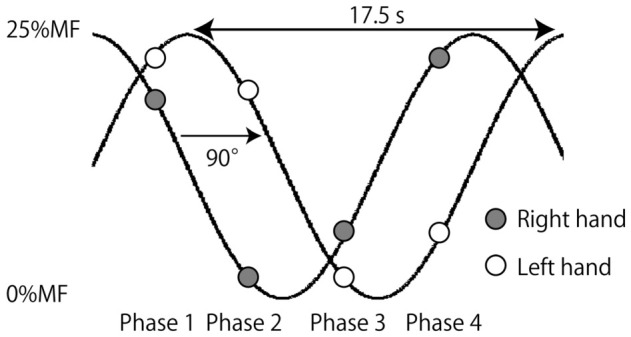
The time course of the force development.

The phases of the tactile stimuli are shown in Figure 2. One cycle was defined as 360 degrees. The position at the 12 o’clock direction was defined as 0 degrees. The movement of the blue circle (left-hand force target) was delayed by 92–93 degrees relative to that of the red circle (right-hand force target).

Tactile stimulation was delivered via vibration applied to a device placed on the dorsal surface of the hand. Stimulation was delivered at four phases. The interval between adjacent phases was 92–93 degrees. The phase of force production in the left hand lagged one phase behind that in the right hand. Thus, the force production phase of the left hand was considered to be delayed by 92–93 degrees relative to that of the right hand.

In phase 1, 2.3 s after the target movement onset along the arc, the stimulation was delivered at 47 degrees and the target coordinates were (165, 113). At this phase, left and right index finger flexion forces were 23% and 20% MF, respectively. In phase 2, 6.8 s after the target movement onset, the stimulation was delivered at 140 degrees and the target coordinates were (113, −165). At this phase, left and right index finger flexion forces were 20% and 2% MF, respectively. In phase 3, 11.3 s after the target movement onset, the stimulation was delivered at 232 degrees and the target coordinates were (−165, −113). At this phase, left and right index finger flexion forces were 2% and 5% MF, respectively. In phase 4, 15.8 s after the target movement onset, the stimulation was delivered at 325 degrees and the target coordinates were (−113, 165). At this phase, left and right index finger flexion forces were 5% and 23% MF, respectively. The timing of stimulation was randomized across trials.

### 2.7. Study Design

Two trial blocks were conducted: one involving bimanual movement with Lissajous feedback (Lissajous feedback task) and the other involving bimanual movement with individual feedback (individual feedback task). The order of the two trial blocks was counterbalanced across participants: eight participants performed the Lissajous feedback task first, whereas seven performed the individual feedback task first. The experiment employed a 2 × 3 within-subject factorial design, with the factors task (Lissajous, Individual) and stimulation (left hand, right hand, no stimulation). Within each trial block, one of the three stimulation conditions (left-hand stimulation, right-hand stimulation, or no stimulation) was assigned to each trial. The order of the stimulation conditions was randomized for each participant using computer-generated random sequences. Ten trials were performed for each stimulation condition at each phase, resulting in 40 trials per block. Participants completed 20 practice trials before each trial block. A 5-min break was provided between the two trial blocks. Participants were not informed about the timing or side of tactile stimulation in advance.

### 2.8. Data Analysis

Force trajectory error was calculated as the difference between target and actual forces within a time window of 100–200 ms after the onset of the vibration stimulus in the no-stimulation condition. The EMG traces were full-wave rectified after baseline correction to zero. The mean amplitude of the full-wave rectified EMG of the FDI within a time window of 100–200 ms after the onset of the vibration stimulus was also calculated (post-stimulus EMG amplitude). This time window fell within the period during which the tactile stimulation lasted 300 ms.

Repeated-measures two-way ANOVA was performed to test the main effects of stimulus (3 levels; left stimulus, right stimulus, and no stimulus) and task (2 levels; Lissajous feedback task and individual feedback task) in each hand in each phase. When Mauchly’s sphericity test was significant, the Greenhouse–Geisser correction was performed for that result whenever it was significant. If there was a significant interaction between the main effects, a test of simple main effects was performed. If there was a significant main or simple main effect, a multiple comparison test (Bonferroni’s test) followed. The alpha level was 0.05. Excel Tokei 2010 ver. 1.13 (Social Survey Research Information, Tokyo, Japan) was used for the statistical analysis.

## 3. Results

### 3.1. Force Trajectory Error

**Figure 3:**
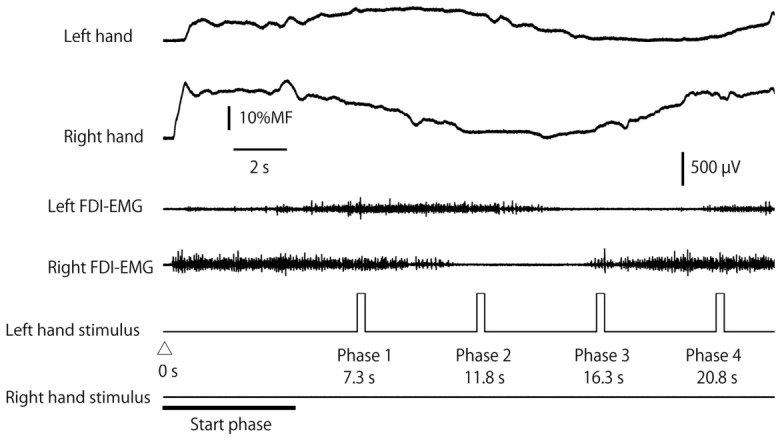
Representative raw traces of force and FDI-EMG during one force production cycle in the Lissajous feedback task.

**Figure 4:**
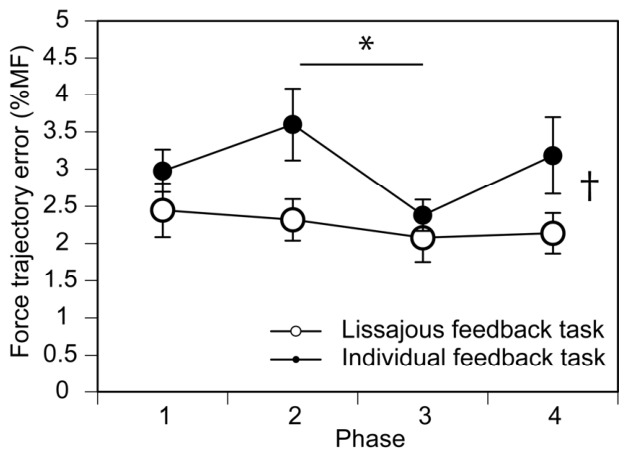
Force trajectory error in each phase, expressed as %MF. Points represent the group mean performance measure for each task and stimulation condition across the four movement phases. Error bars indicate the standard error of the mean (SEM) across participants. An asterisk indicates the significant difference between the phases (*p* < 0.05). A dagger indicates a significant difference between the tasks (*p* < 0.05).

An example of the raw traces of the post-stimulus EMG and the force production during the Lissajous feedback task is shown in Figure 3. The mean force trajectory error in each phase in the individual feedback task is shown in Figure 4. There was no significant interaction between the task and stimulus on the force trajectory error [F(1.859, 26.024) = 2.277, *p* = 0.126, *η*^2^*_P_* = 0.140]. The main effect of the task was significant; the error in the Lissajous feedback task was significantly smaller than that in the individual feedback task [F(1,14) = 18.046, *p* < 0.001, *η*^2^*_P_* = 0.563]. There was a significant main effect of the phase [F(3,42) = 4.192, *p* = 0.011, *η*^2^*_P_* = 0.230]. The multiple comparison tests revealed that the error in phase 3 was significantly smaller than that in phase 2.

### 3.2. Post-Stimulus EMG Amplitude

**Figure 5:**
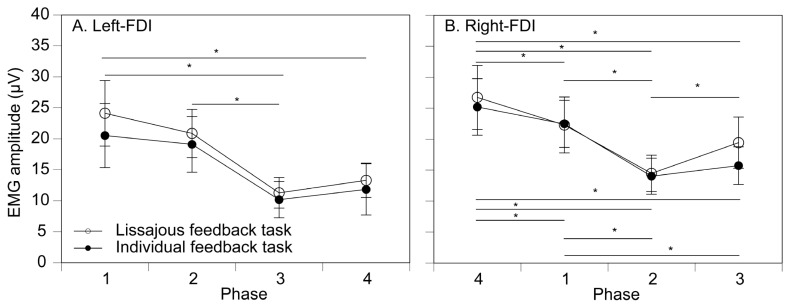
Mean EMG activity during the post-stimulus phase. (**A**) Left FDI and (**B**) right FDI. Data points represent the group mean EMG amplitude measured during the post-stimulus period for each task and stimulation condition across the four movement phases. Error bars indicate the standard error of the mean (SEM) across participants. Asterisks indicate a significant difference between the phases (*p* < 0.05).

The post-stimulus EMG amplitude in the left FDI in the no-stimulation condition is shown in Figure 5A. In the left hand, there was no significant interaction between the task and phase [F(3,42) = 1.132, *p* = 0.347, *η*^2^*_P_* = 0.075]. There was no significant main effect of the task [F(1,14) = 1.006, *p* = 0.333, *η*^2^*_P_* = 0.067]. There was a significant main effect of the phase [F(1.039, 14.551) = 6.706, *p* = 0.020, *η*^2^*_P_* = 0.324]. Multiple comparison tests revealed that the post-stimulus EMG amplitude in phases 3 and 4 was significantly smaller than that in phase 1, and that in phase 3 was smaller than that in phase 2.

The post-stimulus EMG amplitude in the right FDI without the tactile stimulus is shown in Figure 5B. There was no significant main effect of the task [F(1,14) = 1.211, *p* = 0.290, *η*^2^*_P_* = 0.080]. There was a significant main effect of the phase [F(1.411, 19.761) = 15.192, *p* < 0.001, *η*^2^*_P_* = 0.520]. There was a significant interaction between the task and phase [F(3, 42) = 3.490, *p* = 0.024, *η*^2^*_P_* = 0.200]. A test of simple main effect revealed a significant simple main effect of the phase in the Lissajous feedback task [F(3,84) = 13.474, *p* < 0.001, *η*^2^*_P_* = 0.325]. Multiple comparison tests revealed that the post-stimulus EMG amplitude in phase 1 was significantly greater than that in phases 2 and 3, the amplitude in phase 2 was significantly greater than that in phase 3, and that in phase 3 was significantly smaller than that in phase 4 in the Lissajous feedback task. Another test of simple main effect revealed a significant simple main effect of the phase in the individual feedback task [F(3,84) = 14.448, *p* < 0.001, *η*^2^*_P_* = 0.340]. Multiple comparison tests revealed that the post-stimulus EMG amplitude in phases 3 and 4 was significantly smaller than that in phase 1, and that in phases 3 and 4 was significantly smaller than that in phase 2.

### 3.3. Tactile Stimulus Effect on Post-Stimulus EMG

**Figure 6:**
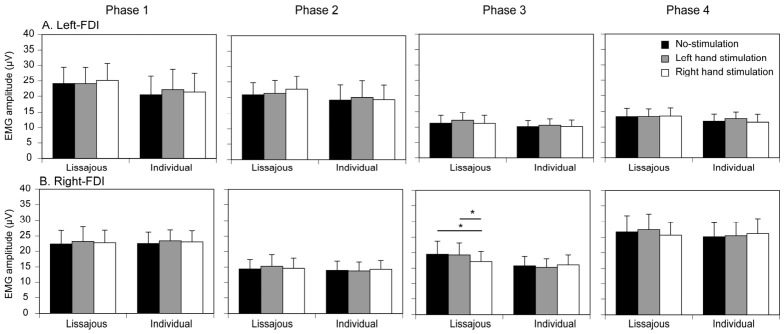
Post-stimulus EMG amplitude under tactile stimulation conditions. (**A**) Left FDI and (**B**) right FDI. Bars represent the group mean post-stimulus EMG amplitude measured within 100–200 ms after stimulus onset for each task and stimulation condition across the four movement phases. Error bars indicate the standard error of the mean (SEM) across participants. Asterisks indicate significant differences revealed by post hoc multiple comparison tests (*p* < 0.05).

The effect of the tactile stimulus and task on the post-stimulus EMG amplitude in the left FDI is shown in Figure 6A. In phase 1, there was no significant interaction between the task and stimulus on the post-stimulus EMG amplitude [F(2,28) = 1.416, *p* = 0.260, *η*^2^*_P_* = 0.092]. There was no significant main effect of the task [F(1,14) = 1.450, *p* = 0.249, *η*^2^*_P_* = 0.094] or stimulus [F(1.383,19.367) = 0.882, *p* = 0.393, *η*^2^*_P_* = 0.000]. In phase 2, there was no significant interaction between the task and stimulus [F(2,28) = 2.981, *p* = 0.067, *η*^2^*_P_* = 0.176]. There was no significant main effect of the task [F(1,14) = 0.759, *p* = 0.398, *η*^2^*_P_* = 0.051] or stimulus [F(2,28) = 1.117, *p* = 0.342, *η*^2^*_P_* = 0.074]. In phase 3, there was no significant interaction between the task and stimulus [F(2,28) = 0.365, *p* = 0.698, *η*^2^*_P_* = 0.025]. There was no significant main effect of the task [F(1,14) = 0.574, *p* = 0.461, *η*^2^*_P_* = 0.039] or stimulus [F(2,28) = 1.895, *p* = 0.169, *η*^2^*_P_* = 0.119]. In phase 4, there was no significant interaction between the task and stimulus [F(1.238,17.330) = 1.435, *p* = 0.255, *η*^2^*_P_* = 0.093]. There was no significant main effect of the task [F(1,14) = 0.622, *p* = 0.443, *η*^2^*_P_* = 0.043] or stimulus [F(2,28) = 1.193, *p* = 0.318, *η*^2^*_P_* = 0.079].

The effect of the tactile stimulus and task on the post-stimulus EMG amplitude in the right FDI is shown in Figure 6B. In phase 1, there was no significant interaction between the task and stimulus [F(2,28) = 0.006, *p* = 0.994, *η*^2^*_P_* = 0.000]. There was no significant main effect of task [F(1,14) = 0.002, *p* = 0.889, *η*^2^*_P_* = 0.001] or stimulus [F(2,28) = 1.392, *p* = 0.265, *η*^2^*_P_* = 0.090]. In phase 2, there was no significant interaction between the task and stimulus [F(2,28) = 0.725, *p* = 0.493, *η*^2^*_P_* = 0.049]. There was no significant main effect of task [F(1,14) = 0.314 *p* = 0.584, *η*^2^*_P_* = 0.022] or stimulus [F(2,28) = 0.105, *p* = 0.901, *η*^2^*_P_* = 0.007]. In phase 3, a significant interaction between task and stimulus was observed [F(2,28) = 3.822, *p* = 0.034, *η*^2^*_P_* = 0.214]. To clarify this interaction, simple main effects were analyzed separately for each task. In the Lissajous feedback task, the tactile stimulus significantly affected the post-stimulus EMG amplitude [F(2,56) = 5.371, *p* = 0.007]. Multiple comparison tests revealed that the EMG amplitude following right-hand stimulation was significantly smaller than that following left-hand stimulation and no-stimulation (both *p* < 0.05; Figure 6B). In contrast, in the individual feedback task, the tactile stimulus did not significantly affect the EMG amplitude. These results indicate that the suppressive effect of right-hand tactile stimulation on the right FDI emerged selectively during the Lissajous feedback task. There was no significant main effect of task [F(1,14) = 3.790, *p* = 0.072, *η*^2^*_P_* = 0.213] or stimulus [F(2,28) = 1.884, *p* = 0.171, *η*^2^*_P_* = 0.119]. In phase 4, there was no significant interaction between the task and stimulus [F(2,28) = 1.357, *p* = 0.274, *η*^2^*_P_* = 0.088]. There was no significant main effect of the task [F(1,14) = 0.788, *p* = 0.390, *η*^2^*_P_* = 0.053] or stimulus [F(2,28) = 0.403, *p* = 0.672, *η*^2^*_P_* = 0.028].

## 4. Discussion

### 4.1. Summary

We hypothesized that separate sensorimotor representations for each hand during bimanual force production with a 90° relative phase would be integrated into a single representation when visual feedback is unified into a single Gestalt via Lissajous feedback of the target force. This integration was expected to result in bilateral suppression of motor output in response to unimanual tactile stimulation. However, unimanual tactile stimulation instead induced the suppression of motor output predominantly in the ipsilateral hand during the Lissajous feedback task. Thus, the present findings do not provide clear support for our hypothesis of a fully shared sensorimotor representation. Rather, they suggest that sensorimotor processing may remain at least partly lateralized and phase-specific even when visual feedback is unified.

The present findings do not necessarily indicate the absence of motor binding during Lissajous feedback. The visual transformation provided by the Lissajous feedback may primarily facilitate performance through perceptual or visuospatial mechanisms, such as Gestalt-based integration of the two target signals [7,8,9,10,11], without requiring a fully shared sensorimotor representation. In this view, visual integration could improve task execution while sensorimotor processing remains partly limb-specific. Future studies are required to dissociate perceptual–visual integration from sensorimotor integration during bimanual coordination.

### 4.2. Masking of Tactile Stimulus-Induced Inhibition

Tactile stimulus-induced suppression of motor output was absent, except in phase 3 of the right hand during the Lissajous feedback task. Corticospinal excitability of hand muscles is suppressed by electrical stimulation of the median nerve delivered approximately 25 ms prior to transcranial magnetic stimulation, a phenomenon known as SAI [21,22]. This inhibitory process is reduced during voluntary movement [26]. Accordingly, SAI may be attenuated during bimanual force production. Tactile gating is known to occur during movement [27,28,29,30,31,32,33,34,35]. Therefore, tactile stimulus-induced suppression of motor output may be masked during bimanual force production due to tactile gating.

### 4.3. Task Difficulty

The tactile stimulus-induced suppression of right-hand motor output during bimanual force production with Lissajous feedback may be related to the facilitated execution of the task due to the integration of the two target signals into a single representation. Error in force production relative to the target force can be used as an index of task difficulty, reflecting the precision of force adjustment. The smaller error observed during bimanual force production with Lissajous feedback in the present study is consistent with previous findings showing that bimanual movement with a 90° relative phase can be performed more smoothly when visual feedback is provided in the form of a Lissajous plot [7,11,12]. This supports the view that visual information from both hands is integrated into a single representation, resulting in motor binding during bimanual movement with a 90° relative phase [14].

In the present study, however, differences in task error between feedback conditions were not phase-dependent. In contrast, tactile stimulus-induced suppression of motor output was phase-dependent: post-stimulus EMG amplitude in the right FDI was smaller than in the other stimulus conditions, particularly in phase 3 during the Lissajous feedback task. Therefore, the facilitated task execution associated with Lissajous feedback does not account for the phase-dependent modulation of tactile stimulus-induced suppression of motor output.

### 4.4. Intermanual Difference

Tactile stimulus-induced suppression of motor output in phase 3 during the Lissajous feedback task was predominantly observed in the hand ipsilateral to the stimulated side. Lissajous feedback has been proposed to integrate visual information from both hands into a single Gestalt [13]. Somatosensory input is primarily conveyed via crossed afferent pathways to the primary somatosensory cortex [36]. Accordingly, the phase-dependent suppression of motor output induced by tactile stimulation during the Lissajous feedback task may reflect the unmasking of ipsilateral motor inhibition due to the integration of visual information from the two hands into a single Gestalt.

SAI is observed in the hand ipsilateral to the motor response side [21,22]. Somatosensory afferent signals are transmitted to the contralateral primary somatosensory cortex [36], indicating that SAI is mediated by crossed afferent pathways. Accordingly, suppression of right-hand motor output induced by right-hand tactile stimulation in the present study may be related to mechanisms similar to those underlying SAI. Previous studies have reported stronger SAI when processed within the left hemisphere, such as right-hand response suppression induced by right median nerve stimulation [37]. Based on this view, the tactile stimulus-induced suppression of particularly the right motor output may be explained by the view that motor binding unmasks sensorimotor interactions within the left hemisphere. Taken together, the ipsilateral suppression of motor output observed during bimanual force production with Lissajous feedback may reflect hemispheric asymmetry in somatosensory-induced motor inhibition, but further studies directly testing hemispheric contributions are required.

### 4.5. Phase Dependency

Tactile stimulus-induced suppression of motor output during the Lissajous feedback task was significant particularly in phase 3. In this phase, post-stimulus EMG amplitude in the left FDI was minimal, indicating that motor output of the left hand was largely suppressed. Motor output of one limb can be suppressed by that of the contralateral limb, a phenomenon known as the bilateral deficit [38]. Accordingly, during the low-output phase of left-hand force production in phase 3, the bilateral deficit affecting the right hand may have been weakest among the phases. Under this condition, tactile stimulus-induced inhibition may have been expressed most clearly in phase 3, because the inhibitory influence from motor commands for the left hand on right-hand motor output via the bilateral deficit was minimal.

### 4.6. Limitations

The generalizability of the present findings should be considered with caution. The sample primarily consisted of right-handed male participants, and it remains unclear whether similar results would be obtained in more gender-balanced samples or in left-handed individuals. In addition, the task involved isometric finger force production under controlled laboratory conditions, which differs from many natural bimanual actions that involve multi-joint movements and dynamic interactions with the environment. Furthermore, the use of the Lissajous feedback represents a highly specific form of visual integration that is not typically available during daily bimanual behavior. Therefore, the present findings may primarily generalize to situations in which bimanual movements are guided by explicit visual representations rather than natural visual or proprioceptive information. Future studies should examine whether similar tactile–motor interactions are observed in more ecologically valid tasks and in more diverse participant populations.

The gender distribution of the participants in the present study was imbalanced (14 males and 1 female). Participants were recruited freely via a website, and no gender-based exclusion criteria were applied; therefore, this imbalance occurred unintentionally. Previous studies have reported gender differences in physical characteristics and motor performance [39,40]. Accordingly, the present findings may have been influenced by the unequal gender distribution of the sample.

A potential limitation of the present study concerns the imbalance in handedness across participants. The majority of participants were right-handed, and only a small number of left-handed individuals were included. Previous research has demonstrated that handedness influences interlimb coupling during bimanual coordination, such that the dominant hand tends to exert a stronger influence on the nondominant hand, resulting in asymmetric interlimb coupling and phase-leading behavior of the dominant limb [41,42]. Given this established asymmetry, the left–right differences observed in the present study may have been partly driven by the predominance of right-handed participants. In particular, the dominant-to-nondominant coupling asymmetry could have contributed to the observed lateralized effects, potentially biasing the group-level results toward patterns characteristic of right-handed coordination dynamics. Therefore, the current findings should be interpreted with caution, as they may not fully generalize to populations with balanced handedness or to left-handed individuals. Future studies should recruit a balanced sample of right- and left-handed participants or explicitly include handedness as a factor in the experimental design. Such an approach would allow the dissociation of task-related lateralization from handedness-related asymmetries in interlimb coordination.

The present findings should be interpreted with caution. First, the observed lateralized suppression does not directly demonstrate hemispheric differences in neural inhibition. The current experimental design did not include neurophysiological measures that would allow for the direct assessment of hemispheric mechanisms. Second, the suppression observed in the right hand during the low-force phase may reflect task-specific factors such as force level, attentional allocation, or biomechanical differences between the hands rather than asymmetries in neural inhibition. Third, the absence of bilateral suppression cannot rule out the possibility that sensorimotor integration occurred at other processing stages that were not captured by the present behavioral and EMG measures.

There was no calibration and reliability information for the intensity of the vibration device. Before the experiment, the experimenter confirmed that the vibration was clearly perceived by the participant. Despite that, in our experience in the preliminary trials, the intensity of the tactile stimulus from the device was weak. Thus, the absence of changes in motor output in most situations observed in the present study may be due to the weak tactile stimulus. This is a limitation of the present study.

## 5. Conclusions

The tactile stimulus to the right hand suppressed the ipsilateral motor output during the low force phase while Lissajous feedback of target force production was provided. The suppression of right-hand motor output induced by right-hand tactile stimulation during the Lissajous feedback task may reflect the unmasking of the tactile stimulus-induced inhibition within sensorimotor processes in the left hemisphere when visual feedback of the two hands is merged into a single representation. The greater suppression during the low-force phase may be due to a weaker bilateral deficit, allowing for easier expression of ipsilateral inhibition. This lateralized tactile stimulus-induced suppression may reflect hemispheric asymmetry in somatosensory-induced motor inhibition.

## Data Availability

The raw data supporting the conclusions of this article will be made available by the authors on request due to or ethical restrictions.

## Abbreviations

The following abbreviations are used in this manuscript:

SAI	Short-latency afferent inhibition
EMG	Electromyography
FDI	First dorsal interosseous muscle
MF	Maximal force
